# Bisphenols, Toxic Elements, and Potentially Toxic Elements in Ready-to-Eat Fish and Meat Foods and Their Associated Risks for Human Health

**DOI:** 10.3390/toxics13060433

**Published:** 2025-05-25

**Authors:** Federica Litrenta, Vincenzo Nava, Angela Giorgia Potortì, Vincenzo Lo Turco, Benedetta Sgrò, Giuseppa Di Bella

**Affiliations:** Department of Biomedical, Dental and Morphological and Functional Imaging Sciences (BIOMORF), University of Messina, Viale Palatucci 13, 98168 Messina, Italy; felitrenta@unime.it (F.L.); angela.potorti@unime.it (A.G.P.); vloturco@unime.it (V.L.T.); benedetta.sgro@studenti.unime.it (B.S.); gdibella@unime.it (G.D.B.)

**Keywords:** bisphenols, bisphenol A diglycidyl ether derivates, intake, metal cans, ready-to-eat food, risk assessment, toxic and potentially toxic elements

## Abstract

In this study, simultaneous exposure to bisphenols and toxic and potentially toxic elements from the consumption of ready-to-eat foods was assessed. In total, 120 different ready-to-eat foods purchased in different Sicilian supermarkets and online shops were analysed. BPA was detected in most of the analysed foods. Statistical analysis was performed, differentiating the samples according to geographical origin and packaging type. Good separation between European and non-European samples could be observed, with the former being characterised by lower levels of Cd and Pb, while the latter showed the highest concentrations of Pb and Cd, followed by Mn and Fe. The calculated estimated weekly intake (EWI) was well below the intake levels recommended by regulatory agencies, indicating that ready-to-eat foods can be safely consumed at expected dietary levels. However, the recently established tolerable daily intake (TDI) for BPA increases the risk quotient values to >1, indicating a risk to the consumer.

## 1. Introduction

Changing consumer lifestyles, characterised by an increasing number of working people, have also led to an increase in the demand for ready-to-eat foods, further fuelling market growth. Ready-to-eat foods offer several advantages to both producers and consumers, including ease of packaging, sterilisation, handling, transportation, and storage. Technological advances in canning and packaging processes have helped improve the quality and safety of canned foods, attracting health-conscious consumers. The global canned food market was estimated to be worth USD 118.53 billion in 2023 and is expected to grow at a CAGR of 3.9% from 2024 to 2030 [1]. Manufacturers are also focusing on sustainable packaging and production practices to satisfy environmentally conscious consumers. Urbanisation and globalisation have also increased the availability and accessibility of canned foods, contributing to the global expansion of the market [1]. Due to the increased frequency of consumption, ready-to-eat meals have become an important part of many people’s diets in today’s society and represent a significant route for potential exposure to various food contaminants [2]. Among the many contaminants present in ready-to-eat foods, bisphenols and their analogues, as well as toxic and potentially toxic elements, represent very topical and extremely important classes of contaminants for the protection of human health. For example, canning is a low-cost method of food preservation that involves heat treatment of canned goods. The use of metal cans should be carefully considered when occasional integrity problems occur due to corrosion that can lead to the migration of metal ions. Metal cans are usually coated with a thin polymer film on the inner surface [3]. However, it is now widely documented that the polymerization of the coating may not be complete and that a significant number of unreacted compounds may be released into the food packaged in coated cans [4,5]. Indeed, migration from packaging materials is a major concern because of the potential adverse health effects. Common materials used to coat food cans are epoxy resins, with possible migration of various bisphenolic compounds [6,7]. Bisphenols, such as Bisphenol A (BPA), are industrial chemicals used in the production of plastics and resins. These materials are commonly found in food packaging, including plastic containers, water bottles, and the inner lining of metal cans. When these materials come into contact with food or drinks, especially under heat or over time, small amounts of bisphenols can leach into the food. This migration can occur more easily with acidic or fatty foods, or when the packaging is damaged or reused. As a result, consumers may be exposed to bisphenols through their diet. In recent years, several studies have been carried out on structural derivates of BPA, such as bisphenol A diglycidyl ether (BADGE), and analogues such as bisphenol S, bisphenol AF, bisphenol F, bisphenol B, etc. These compounds are structural analogues in that they share the basic bisphenol structure on which various functional groups are substituted, or, as in the case of bisphenol A diglycidyl ether (BADGE) and its derivatives, they are basic monomers of epoxy resins (after the reaction of bisphenol A (BPA) with epichlorohydrin) [8]. In fact, BADGE is a key component of epoxy resins; however, migration studies from coated cans have shown that the BADGE migrant undergoes rapid hydrolysis to BADGE·H_2_O and/or BADGE·2H_2_O [8]. Furthermore, when BADGE is used as a scavenger for hydrochloric acid or in the presence of salty foods, BADGE·HCl, BADGE·HCl·H_2_O, and/or BADGE·2HCl are formed [8]. Similar derivatives are obtained when using bisphenol F diglycidyl ether (BFDGE) (synthesised from BPF as an alternative to BPA). The close chemical similarity to BPA has led to the classification of bisphenol analogues as endocrine disrupters. Endocrine disruptors are chemicals that interfere with the body’s hormone system, potentially leading to various health issues. Bisphenol A is a well-studied endocrine disruptor commonly found in food and beverage packaging, raising concerns about potential contamination. The study of bisphenol contamination in food is directly linked to the broader understanding of endocrine disruptors and their potential impacts on human health. Concerns are that BPs may act by interfering with hormone function and potentially cause adverse effects on the reproductive and developmental systems, as well as increase the risk of metabolic disorders such as diabetes and heart disease. However, studies and research into the effects of these bisphenols are still very limited, as is the supporting legislation. It should be noted that BPA and BPS are authorised for use in plastic food contact materials under European Commission Regulation (EU) No 10/2011 and are subject to a specific migration limit (SML) of 0.05 mg kg^−1^. This limit ensures that the food contact material does not pose a risk to human health. It is an essential tool to ensure the health protection of all consumers, including the most sensitive populations. On 19 April, the European Food Safety Authority (EFSA) published a new opinion on BPA (Scientific Opinion—Re-evaluation of the risks to public health related to the presence of bisphenol A in foodstuffs) setting the tolerable daily intake (TDI) at 0.2 ng/kg body weight [9], significantly lower than the 4 µg/kg body weight set in 2015. This change therefore reflects new scientific evidence and aims to improve consumer protection. The new TDI is some 20.000 times lower than its predecessor, making it difficult to stabilise a new, lower SML. As a result, the EU has considered banning its use. In response to the new EFSA opinion, the EU has issued a new regulation setting out specific requirements for BPA, as well as other bisphenol derivatives, regarding their use in the manufacture of materials and articles intended to come into contact with food (December 2024) [10]. At present, the Commission of the European Communities has also set a limit for their migration into food, for the sum of BADGE and its hydrolysed derivatives (BADGE·H_2_O, BADGE·2H_2_O) of 9 mg/kg and for the sum of BADGE formed as reaction products with HCl (BADGE·HCl, BADGE·2HCl, BADGE·H_2_O·HCl) of 1 mg/kg [11]. The Commission also prohibited the use or presence of BFDGE. The new BPA regulation also involves BADGEs as derivatives of bisphenol A [9]. Several toxic and potentially toxic elements are susceptible to migration, including Sn, Fe, Pb, Cd, Cr, Ni, Zn, and Cu, which are considered the most common because they are often used in metal containers [12,13]. In particular, low levels of tin are currently found in foods packaged in unpainted or partially painted tin cans. In addition, Fe is the base element in the steel layer of food cans, while Cd, Ni, and Cu can be found as alloying elements in steel. In addition, Cr treatment is widely used to make the tin layer in tin cans less susceptible to oxidation damage and to improve enamel adhesion. Cr is also commonly used in can ends. Similarly, Zn may be present to improve the corrosion resistance of tin and calcium. Finally, Pb is often found in metal packaging, making it one of the most significant contaminants from packaging materials. European Commission Regulation (EC) No 2023/915 sets maximum levels for certain contaminants, including Pb, Cd, Hg, As, and Sn (inorganic) in many foods [14]. Many factors influence the migration of these organic and inorganic compounds from packaging to food [15]. Previous studies have shown that there is a strong correlation between storage time and the migration of various metals. Significant migration of Sn occurs when cans are not coated with a layer of plastic film; in this case, the high contact temperature appears to accelerate the corrosion of uncoated cans [15]. On the other hand, sterilisation has been shown to have a predominant effect on the migration of bisphenolic compounds. Other factors, such as the nature of the food (pH, fat content, the presence of oxidants and nitrates, etc.), the type of steel, the thickness of the tin coating and surface defects, and the characteristics, thickness, and volume of the paint, can significantly influence the migration of metals and/or bisphenol compounds [16]. The simultaneous exposure risk occurs when an individual is exposed to several potentially toxic chemicals at the same time or in the same context. This type of exposure is common in everyday life but is often underestimated in traditional risk assessment procedures, which tend to consider contaminants one at a time. The risk of simultaneous exposure is therefore an important public health challenge. A cumulative risk assessment is needed that considers not only the individual substances but also their possible interactions. The combined effect, often referred to as the cocktail effect, refers precisely to the phenomenon whereby simultaneous exposure to several chemicals, even at doses that are individually considered safe, can produce unexpected or enhanced toxic effects. This concept is particularly relevant in the case of simultaneous exposure to bisphenols (such as BPA, BPS, BPF) and other chemical contaminants in food. A series of scientific studies document the cocktail effect due to combined exposure to bisphenols and other toxic contaminants; e.g., Evans et al. [17] show that simultaneous exposure to endocrine disruptors during foetal development can cause long-term effects on the endocrine system and neurodevelopment, and that the combination of multiple interferents has effects that cannot be predicted based on a single exposure. Authors Kortenkamp et al. [18] assessed how those mixtures of substances with similar effects act additively or synergistically, even when each is present at levels below the safety threshold. The cocktail effect concept is beginning to influence European and international regulations, but official regulation is still limited, more data are needed, and interdisciplinary cooperation between toxicologists, epidemiologists, regulators, and policy makers is required. With this in mind, this study analysed the presence of bisphenols and toxic and potentially toxic elements in ready-to-eat fish and meat samples consumed worldwide. It also assessed the health risks associated with the combined intake of BPA, Zn, As, Cd, Cu, Mn, Ni, Pb, and Al from the consumption of these foods.

## 2. Materials and Methods

### 2.1. Chemicals and Standard Solutions

Analytical standards of 4,4′-sulfonyldiphenol (BPS), 4,4′-methylenediphenol (BPF), 1,1-bis(4-hydroxyphenyl)ethane (BPE), 4, 4′-(propan-2,2-diyl)diphenol (BPA), 4-[2-(4-hydroxyphenyl)butan-2-yl]phenol (BPB), 2,2-Bis(4-hydroxyphenyl)hexafluoropropane (BPAF), 1,1-bis(4-hydroxyphenyl)-1-phenyl-ethane (BPAP), 1,1-bis(4-hydroxyphenyl)-cyclohexane (BPZ), and 1,4-bis(2-(4-hydroxyphenyl)-2-propyl)benzene (BPP) with a purity of ≥ 99% were purchased from Sigma-Aldrich (Steinheim, Germany). Isotopically labelled standards ^13^C_12_-BPA and ^13^C_12_-BPS, with the purity of ≥99%, were obtained from Cambridge Isotope Laboratories, Inc. (Andover, MA, USA). Standards of bisphenol A diglycidyl ether (BADGE), bisphenol A bis(2,3-dihydroxypropyl) ether (BADGE∙2H_2_O), bisphenol A bis(3-chloro-2-hydroxypropyl) ether (BADGE∙2HCl), bisphenol A (3-chloro-2-hydroxypropyl)(2,3-dihydroxypropyl) ether (BADGE∙HCl∙H_2_O), bisphenol A dimethacrylate (BPADMA), and bisphenol F bis (2,3-dihydroxypropyl) ether (BFDGE∙2H_2_O) were purchased from Fluka (Buchts, Switzerland). The standards, BADGE-D6 and BFDGE-^13^C_12_ were obtained from Sigma-Aldrich (Steinheim, Germany). Acetonitrile (ACN) (HPLC-grade), salts for extraction (sodium chloride (NaCl) and magnesium sulphate (MgSO_4_)), and reagents for purification (primary and secondary amines (PSAs) and sorbent C18) were purchased from Fluka (Milan, Italy).

The reagents used for acid digestion process were ultrapure water, concentrated nitric acid (65%, *v*/*v*), and hydrogen peroxide (30%, *v*/*v*), purchased from J.T. Baker (Milan, Italy). Standard solutions of Al, As, Cd, Cr, Cu, Fe, Mn, Ni, Pb, Sn, and Zn (1000 mg/L in 2% nitric acid) were purchased from Fluka (Milan, Italy) and were used to construct seven-point calibration curves (0.2, 1.0, 2.0, 5.0, 10, 20, and 50 μg/L). Standard solutions of Sc, Ge, In, and Bi (1000 mg/L in 2% nitric acid), used as on-line internal standards at a concentration of 1.5 mg/L, and a standard solution of Re (1000 mg/L in 2% nitric acid), used at a concentration of 0.5 mg/L, were obtained from Fluka (Milan, Italy). Information on BP and BADGE compound names, abbreviations, and validation methods for trace elements are presented in Supporting Information Table S1 and Table S2, respectively.

### 2.2. Sampling

One hundred and twenty samples of ready-to-eat food, including cans, aluminium tubes, plastic boxes, and glass jars of different brands, were purchased in various Sicilian supermarkets and online shops in January 2024. The samples covered two different categories: 90 fish samples, including tuna, mackerel, sardines, salmon, crab, anchovies, clams, shrimp, cuttlefish, and lumpfish, and 30 meat samples, including chicken, beef, and pork. Detailed information on all samples, including origin, type of packaging, ingredients, and fat and protein content, are presented in Table 1 and Table 2.

### 2.3. Sample Preparation

Samples were subjected to QuEChERS extraction for bisphenols, according to the method of Liotta et al. [19]. Briefly, 5 g of sample was weighed and extracted with 5.0 mL ACN. QuEChERS salts (2 g NaCl and 1 g MgSO_4_) were added, and the solution was vortexed and centrifuged. The top layer of ACN (2.0 mL) was transferred to QuEChERS d-SPE (0.25 mg MgSO_4_, 0.1 mg PSA, 0.1 mg C18) and centrifuged again; finally, 1.0 mL of the top layer was filtered through a Millipore PTFE filter (0.22 µm) (Burlington, MA, USA).

For the determination of toxic and potentially toxic elements (Al, As, Cd, Cr, Cu, Fe, Mn, Ni, Pb, Sn, and Zn), samples were subjected to acid digestion using an Ethos 1 microwave system (Milestone, Bergamo, Italy). The operating conditions varied according to the sample category (fish or meat) as described below. The certified reference materials (ERM-CE278k—muscle tissue and ERMBB184—bovine muscle) were also digested under the same conditions as the samples. All determinations were carried out in triplicate. The method proposed by Bruno et al. [20] was used for the mineralization of fish and meat samples. Digested with 8 mL HNO_3_ and 2 mL H_2_O_2_, 0.5 g of each sample was mixed with 1 mL internal Re standard at 0.5 mg/L. Four steps were used in the digestion programme: 10 min at 0–200 °C (step 1), 5 min at constant 200 °C (step 2), 5 min at 200–220 °C (step 3), and 5 min at constant 220 °C (step 4). All steps had a microwave power of 1000 W. After mineralization, each sample was filled with a volume of 25 mL of ultrapure water and filtered through a 0.45 μm filter to remove the larger particles.

### 2.4. Analysis by HPLC-MS/MS

The determination of BP and BAGDE derivatives was performed using an HPLC system (Shimadzu, Kyoto, Japan) coupled to an LCMS-8040 triple quadrupole mass spectrometer with an electrospray ionisation (ESI) source. Data control and analysis were performed using Labsolution software Ver. 6.115. Chromatographic separation was performed for both BPs and BADGEs on an Agilent Zorbax SB-C18 column (Santa Clara, MA, USA). (5 µm, 4.6 × 250 mm). The method of Liotta et al. [19] was used for BPs (BPS, BPF, BPE, BPA, BPB, BPAF, BPAP, BPZ, BPP). The flow rate was 0.7 mL/min. The mobile phases were ultrapure water (solvent A) and acetonitrile (solvent B). The following linear gradient was used: 0–7 min, 20–40%B; 7–25 min, 40–90%B; and 25–35 min, 90–20%B. Thus, the total separation time was 25 min. Figure S1 shows an example of a chromatogram obtained for one of the analysed samples. The compounds BADGE, BADGE∙2H_2_O, BADGE∙2HCl, BADGE∙HCl∙H_2_O, BPADMA, and BFDGE∙2H_2_O were determined according to the method of Yonekubo et al. [21]. The mobile phases were methanol with 5 mM ammonium acetate. The flow rate was 0.25 mL/min. The chromatographic run was 10 min as follows: 0–1 min, 25% methanol, 1–5 min, 25–75% methanol, and 5–10 min, 75% methanol. BADGE and its derivatives showed a high tendency to form [M + NH_4_]^+^ ionic adducts with the mobile phase components. Therefore, 5 mM ammonium buffer was used as a modifier in the mobile phase, allowing for the formation of ammonium adducts and ensuring signal reproducibility. The injection volume for both methods was 20 µL. For ESI-MS, mass spectrometric analyses were performed for BPs in negative ion mode (M − H^−^), and for BADGE derivatives they were performed in positive ion mode ([M + NH_4_]^+^). Figure S2 shows an example of an ESI mass spectrum obtained for one of the samples. The MS operated under the following conditions: nebulizing gas flow 3.0 L/min, nebulizing gas pressure 770 KPa, drying gas flow 15.0 L/min, DL temperature 250 °C, and CID gas 230 KPa. Multiple reaction monitoring (MRM) was used for all quantitative and qualitative data. Full details are given in Table S1. The method was evaluated for sensitivity (limit of detection (LOD) and limit of quantification (LOQ)), linearity (R^2^), and accuracy (% recovery), as reported in Supporting Information (Table S1). The linearity was assayed by analysing six mixtures of standard solutions in the range of 1.0–500 μg/kg.

### 2.5. Analysis by ICP-MS

Al, As, Cd, Cr, Cu, Fe, Mn, Ni, Pb, Sn, and Zn contents were determined using an iCAP-Q ICP-MS spectrometer (Thermo Scientific, Waltham, MA, USA). The ICP-MS characteristics and operating conditions were the same as those reported in our previous work. The monitored isotopes were ^27^Al, ^52^Cr, ^55^Mn, ^56^Fe, ^60^Ni, ^63^Cu, ^66^Zn, ^75^As, ^114^Cd, ^120^Sn, and ^208^Pb. Integration times were 0.5 s/point for Fe and As and 0.1 s/point for other elements. All samples were analysed in triplicate.

The method was evaluated according to Eurachem criteria for sensitivity (LOD and LOQ), linearity (R^2^), and accuracy (% recovery), as shown in Table S2. Linearity was determined by linear least squares regression and seven-point calibration curves (range: 0.2–50.0 μg/kg).

### 2.6. Statistical Analysis

All statistical calculations were performed using SPSS 13.0 software for Windows (SPSS Inc., Chicago, IL, USA). The initial multivariate matrix was made up of 120 cases (90 ready-to-eat fish samples and 30 ready-to-eat meat samples) and 12 variables (BPA, Zn, As, Cd, Cr, Cu, Fe, Mn, Ni, Pb, Sn, and Al determined in the samples analysed). The second and third data sets each consisted of a matrix of 90 cases (only fish samples) and the same 12 variables as in the first data set; in the second data set, the cases were divided into two groups according to geographical origin (European and non-European samples), while in the third data set, the cases were divided into four groups according to packaging type (can, aluminium collapsible tube, plastic box, and glass).

First, a one-way ANOVA with the Kruskal–Wallis post hoc multiple comparison test was used to assess the significance of differences among all samples of different types; second, the non-parametric Mann–Whitney U test was used to assess the significance of differences between fish samples according to geographical origin. In the third step, a one-way ANOVA with the Kruskal–Wallis post hoc multiple comparison test was performed to check the differences according to packaging type. For each variance examined, statistical significance was accepted at *p* < 0.01.

The second and third data sets were then normalised to obtain the independence of the scaling factors of the different variables. After checking the suitability of the initial data using the Kaiser–Meyer–Olkin (KMO) test and Bartlett’s test, the data were subjected to Principal Component Analysis (PCA) in order to try to differentiate the samples according to both geographical origin and type of packaging.

### 2.7. Consumer Health Risk Estimation

Estimated weekly intake (EWI) and hazard quotient (HQ) values were calculated to assess the health risks to consumers associated with the consumption of ready-to-eat foods. It was assumed that these foods are consumed twice a week and that a 70 kg individual consumes an average of 75–120 g per day. For canned foods, the amount indicated on the label was used, as many of these can be considered a full meal; for other foods, an amount of about 100g was used. The EWI was calculated taking into account the concentration of the analyte found, weekly consumption, body weight and different tolerable daily intake (TDI), tolerable weekly intake (TWI), provisional tolerable weekly intake (PTWI) and benchmark dose, and lower confidence limit (BMDL). In April 2023, EFSA published a scientific opinion re-evaluating the public health risks associated with the presence of BPA in food and setting a new TDI of 0.2 ng BPA/kg body weight [9]. EFSA also established a weekly intake (TWI) of 1 mg/kg body weight for Al [22], a tolerable daily intake (TDI) of 13 μg/kg bw for Ni [23], a weekly intake (TWI) of 2.5 µg/kg body weight for Cd [24] and a BMDL01 of 0.5 µg/kg for Pb [25]. For Zn, the Scientific Committee on Food indicates a tolerable upper limit of 7 mg/d; for Mn, Codex Alimentarius Commission standard limit of 2.5 mg/kg PTWI; for Cu PTWI of 3.5 mg/kg; and for As, PTWI limit of 0.015 mg/kg [26].

## 3. Results and Discussion

### 3.1. Occurrence of Bisphenols and Elements in Samples

The HPLC-MS/MS method was used to analyse bisphenols and BADGEs derivatives in ready-to-eat foods. A total of 120 samples of meat and fish were analysed, and the results are presented in Table 3 and Table 4. BPA was present in 70% of the fish samples with levels ranging from 2.68 to 47.69 µg/kg. BPF was present in 17% of the samples, followed by BPB, which was present in only 11% of the samples. In meat samples (Table 4), BPA was present in 90% of the samples, followed by BPF and BPB at 40% and 20%, respectively. The bisphenols BPS, BPE, BPZ, BPP, BPAP, and BPAF were not found in any sample. With regard to BADGE derivates in fish samples, BADGE was present in 42% of the samples, while its hydrolysis and chlorination products BADGE∙2H_2_O, BADGE∙HCl∙H_2_O, and BADGE∙2HCl were present in 37%, 32%, and 23% of the samples, respectively. In the processed meat samples, BADGE was present in 50% of the samples, followed by BADGE∙2H_2_O (40%), BADGE∙HCl∙H_2_O (20%), and BADGE∙2HCl (20%). The levels of BP analogues and BADGEs were all below the limit of quantification in samples of canned Atlantic mackerel fillets, natural Alaskan salmon, salmon pate, anchovy paste in olive oil, shelled clams, natural shrimps, lumpfish eggs, cuttlefish ink, canned halal chicken, canned beef, and pork pate. With the exception of samples of salmon pate, which had a lipid content of 13.5%, all the other samples mentioned above had a relatively low lipid content (between 0.1% and 5.2%). Significantly lower concentrations (*p* < 0.01) of BPA were found in canned sardines in olive oil (2.85 µg/kg) from Morocco, dried shrimp (2.68 µg/kg) from Argentina, and canned beef with gelatine (0.52 µg/kg) from Italy, whereas samples of canned crab meat in brine (47.69 µg/kg) from Indonesia and canned chicken breast with jelly (29.57 µg/kg) from Italy had significantly higher concentrations of BPA. The fish samples differed not only in terms of fish species and different fat content but also in terms of packaging type—can, plastic box, aluminium collapsible tube, and glass jar. With regard to the type of packaging, the canned samples contained BP analogues and BADGEs. In particular, the sample with the most compounds (BPs and BADGEs) was the canned mackerel in *sos tomat* from Romania, followed by the canned crab meat in brine from Indonesia. The canned mackerel in *sos tomat* contained, in addition to BPA (12.09 µg/kg), concentrations of BPF (24.06 µg/kg), BPB (36.16 µg/kg), BADGE (361.54 µg/kg), BADGE∙2H_2_O (199.66 µg/kg), BADGE∙HCl∙H_2_O (20.86 µg/kg), and BADGE∙2HCl (17. 25 µg/kg), while the canned crab meat in brine contained, besides the highest level (compared to all samples) of BPA (47.69 µg/kg), residues of BPF (10.09 µg/kg), BADGE (28.70 µg/kg), BADGE∙2H_2_O (12.44 µg/kg), BADGE∙HCl∙H_2_O (37.14 µg/kg), and BADGE∙2HCl (15.15 µg/kg). No significant levels of bisphenol A analogues and BADGE derivates were found in samples packaged in plastic boxes, aluminium collapsible tubes, and glass jars. The only exceptions were surimi sticks, which contained BADGE (55.85 µg/kg), BADGE∙2H_2_O (12.88 µg/kg), BADGE∙HCl∙H_2_O (36.71 µg/kg), and BADGE∙2HCl (15.52 µg/kg) in addition to BPA, and dried sardines, which contained BADGE at a concentration of 90.27 µg/kg. The meat samples were all canned, which is why it was not possible to make a comparison with different types of packaging. The samples with the highest levels of bisphenol A analogue residues and BADGEs were the canned minced pork and ham with bacon samples, in which BPA (9.17 µg/kg), BPB (2.34 µg/kg), BADGE (151.02 µg/kg), BADGE∙2H_2_O (201.90 µg/kg), BADGE∙HCl∙H_2_O (23.14 µg/kg), and BADGE∙2HCl (13.81 µg/kg) were present, and the canned luncheon chicken meat, which contained levels of BPA (25.12 µg/kg), BADGE (100.62 µg/kg), BADGE∙2H_2_O (9.94 µg/kg), BADGE∙HCl∙H_2_O (10.75 µg/kg), and BADGE∙2HCl (22.86 µg/kg) (see Table 4). The meat samples that had significantly lower BPA values were canned cooked ham, canned beef with gelatine, canned beef and pork pâté, and canned meat broth, with concentrations ranging from 0.52 µg/kg to 4.30 µg/kg. Given these results, it is clear that canned foods are highly affected by bisphenol migration; this could be due to the can sterilisation process, which may favour migration, as confirmed by other studies [27,28,29]. The concentrations of bisphenols and BADGE derivatives are highly variable as they depend on so many factors. Compared to the context of the literature, the results of this study are in line with other studies. For example, Choi and colleagues [30] found concentrations of BPA ranging from 1.41 to 278.50 µg/kg and concentrations of BADGE derivatives as high as 1525.00 µg/kg in samples of canned foods, including meat, ham, fish, vegetables, and fruit. In another study, Cao et al. [31] found BPA, BPF, and BPS in canned foods, including meat, seafood, mushrooms, fruits, and vegetables, with concentrations ranging from 0.3 to 837 µg/kg for BPA, 0.3 to 75.4 µg/kg for BPF, and 0.05 to 1.16 µg/kg for BPS. In a study conducted in Portugal on samples of canned meat, BPAF, BPF, and BPB were found in addition to BPA; BPA levels ranged from no evidence to a maximum of 202 µg/kg, with a mean of 42 µg/kg; and for the analogues, levels were found to be 5.9 to 36.5 µg/kg for BPB, 9.6 to 153 µg/kg for BPF, and 4.3 to 13 µg/kg for BPAF [32]. Lestido-Cardama et al. [33] found BADGE derivatives ranging from <0.5 to 724 µg/kg in canned foods, including fish, seafood, vegetables, cereals, and fruits.

The concentrations of toxic and potentially toxic elements are shown in Table 5 and Table 6. In fish samples (see Table 5), the most representative trace elements were Zn and Fe, followed by Al, Cu, and Mn. Significantly higher values (*p* < 0.01) of Zn were found in the samples of canned sardines in olive oil, canned sardines in vegetable oil, and dried sardines, with concentrations ranging from 22.83 to 26.19 mg/kg, while significantly lower values were found in surimi sticks and natural shrimp. Samples of shelled clams had the highest Fe values (70.41 mg/kg), followed by samples of anchovy paste in olive oil (33.17 mg/kg) and dried sardines (30.18 mg/kg). Significantly lower levels of Fe were found in the samples of surimi sticks (3.90 mg/kg). The trend for Al was particularly interesting; all canned samples showed concentrations ranging from 1.97 µg/kg in the canned crab meat in brine samples to 8.54 mg/kg in the canned natural grilled mackerel fillet samples. The remaining samples had significantly lower Al concentrations (*p* < 0.01), with the exception of salmon pâté (3.37 mg/kg) and anchovy paste in olive oil (7.68 mg/kg)—the latter being the only two samples in the aluminium collapsible tube. Significantly lower Cu levels were found in samples of canned crab meat in brine (0.46 mg/kg), and significantly higher levels (*p* < 0.01) were found in dried shrimp (13.46 mg/kg). Mn concentrations ranged from a minimum of 0.05 mg/kg in canned pink salmon to a maximum of 2.46 mg/kg in samples of dried sardines. For Pb, all values were within the legal limits set by EU Commission Regulation 2023/915 of 25 April 2023 on maximum levels of certain contaminants in food [14]. Concentrations ranged from 0.03 mg/kg in canned natural tuna to 0.21 mg/kg in salmon pâté samples. Sn concentrations were also in line with the stable limits for canned food and ranged from 0.04 mg/kg in samples of natural shrimps to 0.84 mg/kg in samples of natural Alaskan salmon. EU Regulation 2023/915 also sets the maximum limit for Cd in the muscle of mackerel and tuna at 0.10 mg/kg, in the muscle of anchovy, swordfish, and sardine at 0.25 mg/kg, in crustaceans at 0.50 mg/kg, and in all other fish at 0.05 mg/kg. All fish samples analysed showed concentrations within the legal limits for Cd.

The toxic and potentially toxic elements of meat samples are shown in Table 6. Again, the variability of the elements is very high; the most representative trace element was Fe, followed by Zn, Al, and Mn. Significantly lower values of Fe were found in samples of canned light chicken and canned halal chicken with concentrations of 10.56 mg/kg and 11.23 mg/kg, respectively; the highest values of Fe were found in samples of canned beef with gelatine (27.95 mg/kg). Zn was significantly lower in canned cooked ham samples with a concentration of 6.87 mg/kg, while canned meat broth samples had significantly higher Zn concentrations (19.54 mg/kg). Al ranged from a minimum of 0.54 mg/kg in canned beef and pork pâté to a maximum of 4.90 mg/kg in canned luncheon chicken meat samples. For Pb and Cd, EU Regulation 2023/915 sets a limit of 0.10 mg/kg for Pb and 0.05 mg/kg for Cd in beef, pork, and sheep meat. Canned halal chicken, canned meat broth, canned beef and pork pâté, canned luncheon chicken meat, canned cooked ham, canned beef with gelatine, and canned beef and pork pâté samples exceeded the maximum limit for Pb, while canned minced pork and ham with bacon samples exceeded the maximum limit for Cd. The samples of canned horse mackerel, canned smoked mackerel fillets, mackerel in *sos tomat*, and canned pink salmon, in addition to having the highest concentrations of BADGE derivatives, also had high concentrations of Al, Fe, Sn, and Pb. This was probably due to the fact that the cans used for packaging were of poor quality or damaged, allowing for the simultaneous migration of both bisphenols and toxic and potentially toxic elements [29].

As confirmed by other studies [5,34,35], toxic and potentially toxic elements may be present in packaged and canned food as a result of environmental contamination of the food or by migration from the packaging material. Metal food packaging is mostly composed of tinplate (tin-coated steel), chromium-coated steel, or aluminium, mostly coated internally with a resin to protect the food from contact with the metal. However, when metal is exposed to food due to damage of the coating, corrosion is accelerated, and elements such as Sn, Fe, Cd, and Pb can be released, increasing their levels in food [34]. Container materials and food ingredients all play a role in the levels of BPA and other harmful elements in food and drink. These can leach into food from plastic containers and canning liners, especially when heated or scratched. Co-existing ingredients and foods can also affect the migration of BPs and toxic elements, with fats and acidic foods increasing it. Research consistently shows that storage time, temperature, and the physical conditions of food containers are critical factors that influence the extent to which bisphenols migrate into food or beverages [34,35]. Migration is a time-dependent process, and prolonged storage, even at room temperature, can lead to a gradual build-up of the chemicals in food. This is particularly important for canned foods, which often have a long shelf life. High temperatures greatly accelerate the rate of migration. Heat can increase the mobility of molecules and weaken chemical bonds in container materials such as polycarbonate plastics and epoxy coatings, facilitating release [35].

### 3.2. PCA Results

**Figure 1:**
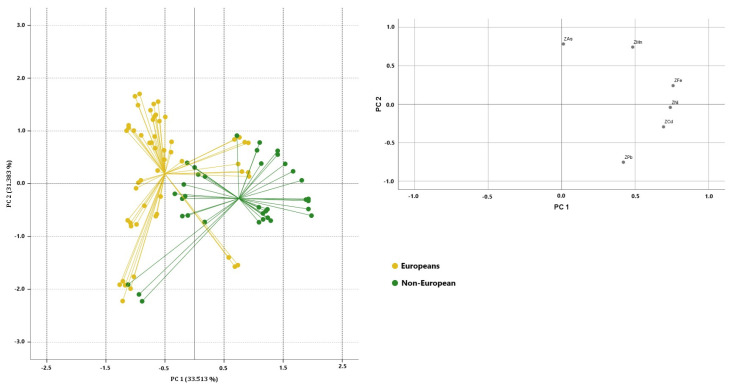
Two-dimensional scatter plots and loading graph for ready-to-eat fish samples categorised by geographical origin (European versus non-European samples). Inset: load plot for PC1 and PC2.

**Figure 2:**
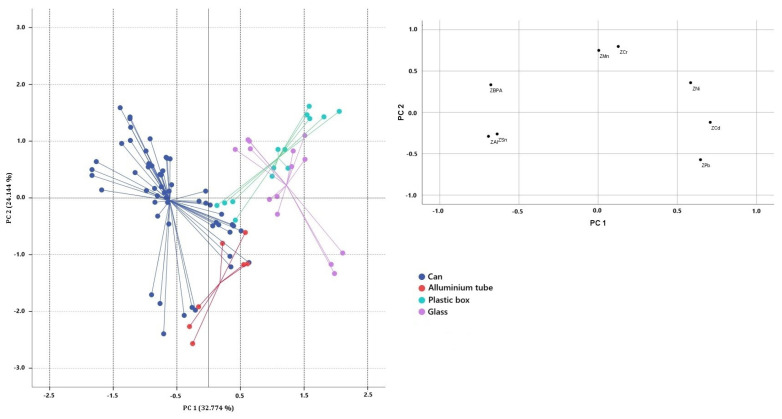
Two-dimensional scatter plots and loading graph for ready-to-eat fish samples categorised by type of packaging. Inset: load diagram for PC1 and PC2.

PCA was applied to the two normalised data sets, using the concentrations of bisphenols and toxic/potentially toxic elements found to be significantly different as variables in order to differentiate the samples according to geographical origin (significantly different: As, Cd, Fe, Mn, Ni, and Pb) and packaging type (significantly different: BPA, Cd, Cr, Mn, Ni, Pb, Sn, and Al). The suitability of the data for factor analysis was checked. In the first data set (European, non-European), the Kaiser–Meyer–Olkin (KMO) sampling adequacy measure gave a value of 0.548, and Bartlett’s sphericity test gave an approximate chi-square value of 141. 338 (at a *p*-level below 0.000), while for the second data set (packaging type) the KMO sampling adequacy measure gave a value of 0.434 and Bartlett’s sphericity test gave a chi-square value of 266.100 (at a *p*-level below 0.000). The two correlation matrices were then factored and fitted to the respective PCAs. The analysis of the first correlation matrix (Figure 1) shows that the highest positive correlations were observed for Pb-Cd (0.520), Mn-As (0.474), and Ni-Fe (0.448), while the highest negative correlations were observed for Pb-As (−0.376) and Pb-Mn (−0.324). According to the Kaiser–Guttman criterion, two principal components with eigenvalues greater than one (2.011 and 1.883) were extracted, which together explain 64.896% of the total variance (33.513% and 31.383%, respectively). The extracted components were able to reproduce all variables well, as there were no variables with low saturation in each factor, and the commonality was always above 0.633. The first component has the highest positive correlation with As and Mn, while negative correlations are observed for Pb and Cd; Figure 1 shows the 2D scatterplot on the plane defined by PC1 and PC2 for the fish samples categorised by geographical origin. On the first component, good separation between the European and non-European samples can be observed; the European samples are characterised by lower values of Cd and Pb, whereas the non-European samples show the highest concentrations of Pb and Cd, followed by Mn and Fe, with the exception of the canned pink salmon samples, which showed low concentrations of these elements. The analysis of the second correlation matrix shows that the highest positive correlations were observed for Pb-Cd (0.520), Mn-Cr (0.395) and Sn-BPA (0.391), and Al-Sn (0.368), while the highest negative correlations were observed for Al-Ni (−0.600), Pb-BPA (−0.489), and Sn-Cd (−0.397). Four principal components with eigenvalues greater than one (2622, 1932, 1055, and 1000) were extracted, which together explain 82.608% of the total variance (32.774%, 24.144%, 13.190%, and 12.500%, respectively) as is shown in Figure 2. As there were no variables with low saturation in each factor and the commonality was always above 0.652, the extracted components were able to reproduce all variables well. The canned samples were separated from the other samples on PC1 and had strong positive correlations with BPA and strong negative correlations with Al and Sn, except for the samples canned horse mackerel, canned sardines in olive oil, and canned sardines in vegetable oil. The samples with plastic and glass packaging are located in the positive PC2 quadrants and show strong positive correlations with Cr and Ni and strong negative correlations with Pb and Cd.

### 3.3. The Uptake of Bisphenols and Toxic/Potentially Toxic Elements from Processed Food

To assess the health risks of the consumption of processed foods contaminated with BPA and toxic and potentially toxic elements, the estimated weekly intake (EWI) was calculated, and the hazard quotient (HQ), which is the ratio of a given EWI to the corresponding reference dose, was also used to assess the plausibility of the risk. An HQ (dimensionless) > 1 indicates a high risk. The EWIs and HQs calculated in accordance with Storelli et al. [36] are shown in Table 7.

The results show that the calculated EWIs for Zn, As, Cd, Cu, Mn, Ni, Pb, and Al were well below the intake levels of the relevant values recommended by regulatory agencies, indicating that the processed food can be safely consumed at the intended dietary levels. For the risk assessment, the HQ in this case did not exceed the threshold value of 1 for each toxic and potentially toxic element that could be consumed by adults, indicating that the adverse health effects of consuming these foods are not significant.

A different discussion should be made for BPA; as can be seen in the table, the EWIs calculated for BPA exceeded the EFSA dose in all samples by a considerable amount. EWI values ranged from a minimum of 6.13 µg/kg in dried shrimp to a maximum of 116.62 µg/kg in grilled mackerel fillets in olive oil, covering a risk quotient ranging from 4.38 to 83.30. EFSA’s review of BPA resulted in a TDI of 0.2 ng/kg bw per day, replacing the previous provisional level of 4 µg/kg bw. The new TDI was therefore reduced by 20,000. At this new level, all HQs calculated for the samples analysed were >1 and therefore pose a risk to consumers.

## 4. Conclusions

This work highlights the simultaneous migration of toxic and potentially toxic elements and bisphenol compounds from different types of ready-to-eat food consumed worldwide. BPA is the most abundant bisphenol analogue in all samples; BPF and BPB analogues were found as substituted monomers of BPA. Concentrations of BADGE derivatives were mainly found in products in cans compared to those in aluminium tubes and glass jars. A strong correlation was observed between Pb and BPA and between Sn and BPA and between Al and Sn in canned products, probably due to can damage. The intake of bisphenols and toxic/potentially toxic elements from canned/packaging foods showed that the calculated EWIs for Zn, As, Cd, Cu, Mn, Ni, Pb, and Al were well below the intake levels of the relevant values recommended by the regulatory authorities, while the calculated EWIs for BPA were well above the EFSA dose in all samples. Consequently, all calculated HQs for the analysed samples were >1 and therefore posed a risk to consumers. On 9 February 2024, the European Commission launched a public consultation on the use of bisphenol A together with other bisphenols, which ended with the publication of the new regulation (Commission, 2024) banning the use of BPA and its derivatives in food contact materials. To prevent BPA from being replaced by equally dangerous substances, the new regulation extends its caution to all other bisphenols used in food contact materials, which will have to be evaluated and authorised before use to ensure they do not pose a risk to human health. This new regulation, which will come into force on 20 July 2026, represents a challenge for the packaging industry and opens the door to a new era in which food safety and sustainability are priorities.

## Figures and Tables

**Table 1:** List of processed fish samples analysed.

Sample	N°	Origin	Packaging Type	Protein (%)	Fat (%)	Ingredients	Manufacture Data/Expired Data
Canned mackerel fillets	4	Portugal	Can	24	4.9	Mackerel fillets, olive oil, salt.	*/11 November 2026
Canned horse mackerel	4	Chile	Can	23	6.2	Mackerel, water, salt.	*/24 May 2025
Grilled mackerel fillets in olive oil	4	Portugal	Can	23	21	Mackerel (87%), organic extra virgin olive oil, salt.	*/October 2024
Canned smoked mackerel fillets	4	Ireland	Can	20	22	Mackerel fillet (73.5%), sunflower oil, black pepper, salt.	*/September 2024
Natural grilled mackerel fillets	3	Portugal	Can	20	9.4	Mackerel (87%), water, salt.	*/November 2024
Mackerel in *sos tomat*	3	Romania	Can	8.4	13.6	Mackerel with skin, tomato sauce, water, tomato paste, sugar, rapeseed oil, potato flour, salt, dried onion, xanthan gum, dried parsnips, spices, spice extracts (celery, pepper), acetic acid.	*/August 2024
Canned Atlantic mackerel fillets	3	Morocco	Can	25	3	Mackerel, Olive Oil, Salt.	*/November 2024
Canned tuna in olive oil	4	Italy	Can	22	2.4	Tuna, olive oil, salt.	*/September 2025
Canned natural tuna	4	Italy	Can	24.4	1.2	Tuna, water, salt.	*/March 2024
Canned tuna pâté	4	Italy	Can	13	15	Tuna (37%), water, corn seed oil, green olives, soya protein, modified maize starch, capers, salt, oregano, natural flavouring nutmeg with other natural flavourings.	*/February 2025
Natural Alaskan salmon	3	Denmark	Can	22.7	2.3	Salmon, water, salt.	*/31 December 2025
Canned pink salmon	3	USA	Can	19	7	Salmon, water, salt.	*/December 2024
Canned sardines in olive oil	4	Morocco	Can	16	9	Sardines, olive oil, salt.	7 February 2019/April 2024
Canned sardines in vegetable oil	4	Morocco	Can	23.9	17	Sardines, vegetable oil, salt.	*/September 2024
Canned crab meat in brine	4	Indonesia	Can	12	10	Crab meat, water, salt, sugar, acidifier (E330), sodium metabisulphite, (E385).	*/March 2024
Salmon pâté	3	Italy	Aluminium collapsible tube	18.5	13.5	Pink salmon (46%), water, corn oil, rice starch, flavourings, salt, paprika extract.	*/15 March 2026
Surimi sticks	4	Estonia	Plastic box	7	4.3	Surimi 40%, water, potato starch, egg white, rapeseed oil, salt, sugar, flavours, soy, carrageenan, monosodium glutamate, titanium dioxide, paprika oleoresin, carmine.	*/20 July 2025
Anchovies paste in olive oil	4	USA	Aluminium collapsible tube	17.7	11	Anchovies, salt, olive oil, palm oil, sugar, spices, sodium benzoate.	*/September 2024
Shelled clams	4	Italy	Glass	10	1.5	Clams and their natural broth, water, salt, citric acid.	*/June 2025
Natural shrimp	4	Italy	Glass	8	1.5	Peeled and pre-cooked shrimps, water, salt, sugar, citric acid. May contain traces of shellfish and fish.	*/February 2025
Lumpfish eggs	3	Denmark	Glass	11	3.3	Lumpfish eggs (90%), water, salt, stabiliser (415), colouring agents (150d, 151, 110), acidity regulator.	*/February 2025
Cuttlefish Ink	3	Spain	Glass	7.3	0.1	Cuttlefish ink, water, salt, thickener: carboxymethylcellulose.	*/16 March 2025
Dried sardines	5	Argentina	Plastic box	7	5.1	Sardines.	*/October 2024
Dried shrimp	5	Argentina	Plastic box	6	4.5	Shrimp.	*/December 2024
**Total**	**90**						

Can: canned food; * production date was not available.

**Table 2:** Canned and non-canned meat food samples analysed.

Sample	N°	Origin	Packaging Type	Protein (%)	Fat (%)	Ingredients	Manufacture Data
Canned light chicken	3	Italy	Can	11	13.5	Chicken, salt.	*/20 February 2025
Canned halal chicken	3	Poland	Can	13	4.7	Chicken, salt (2%), sugar, cooked chicken powder, sodium phosphates, spices, sodium erythorbate, sodium nitrite, natural flavouring.	*/March 2025
Canned meat broth	3	Italy	Can	11	1.2	Beef stock, salt, natural flavouring, yeast extract, Mirepoix (carrots, celery, onions), brown sugar.	23 March 2020/February 2024
Canned beef and pork pâté	3	Italy	Can	11	5.2	Pork, beef, stock, salt, spices.	*/October 2025
Canned chicken breast with jelly	3	Italy	Can	11	19	Cooked chicken breast stock 38%, agar-agar gelling agent, locust bean gum, monosodium glutamate, water, salt, flavourings.	*/September 2024
Canned luncheon chicken meat	3	Philippines	Can	12	10	Chicken, salt.	*/June 2026
Canned cooked ham	3	Italy	Can	9	26	Cooked ham (37%), water, salt, lactose, maize seed oil, soya protein, maize starch, locust bean gum, flavourings.	12 January 2023/February 2024
Canned beef with gelatine	3	Italy	Can	11	1.5	Cooked beef, beef, water, salt, sugar, sodium nitrite.	20 September 2023/April 2024
Canned beef and pork pâté	3	Italy	Can	11	14	Pork, beef, stock, salt, spices.	*/February 2026
Canned minced pork and ham with bacon	3	Denmark	Can	15	22	Pork (75%), smoked bacon, salt, water, antioxidants (E301), preservatives (E251), starch, ham 2%, sugar, stabilisers (E450), spice extract.	*/October 2026
**Total**	**30**						

Can: canned food; * production date was not available.

**Table 3:** Bisphenols and BADGE derivates (expressed in µg/kg) of processed fish samples. Data are expressed as mean ± standard deviation, and Kruskal–Wallis test statistics are reported.

Samples	BPA	BPF	BPB	BADGE	BADGE∙ 2H_2_O	BADGE∙HCl∙ H_2_O	BADGE∙ 2HCl
Canned mackerel fillets	20.36 ± 0.82 ^c^	-	-	20.37 ± 0.94 ^a^	39.76 ± 1.08 ^b^	34.31 ± 1.00 ^b,c^	9.99 ± 0.68 ^a^
Canned horse mackerel	4.36 ± 0.36 ^a^	70.39 ± 0.66 ^c^	-	100.03 ± 1.03 ^c^	-	-	-
Grilled mackerel fillets in olive oil	34.02 ± 0.73 ^c,d^	-	-	30.30 ± 0.83 ^a^	20.81 ± 0.50 ^a^	40.05 ± 0.48 ^c^	-
Canned smoked mackerel fillets	31.85 ± 0.86 ^c,d^	-	9.58 ± 0.04	102.13 ± 2.91 ^c^	50.92 ± 0.30 ^b^	-	-
Natural grilled mackerel fillets	9.31 ± 0.13 ^b^	-	-	20.61 ± 0.33 ^a^	15.93 ± 0.18 ^a^	6.44 ± 0.42 ^a^	78.70 ± 0.97 ^c^
Mackerel in *sos tomat*	12.09 ± 0.16 ^b^	24.06 ± 0.57 ^b^	36.16 ± 0.70	361.54 ± 36.47 ^d^	199.66 ± 1.70 ^c^	20.86 ± 0.55 ^b^	17.25 ± 0.07 ^b^
Canned Atlantic mackerel fillets	-	-	-	-	-	-	-
Canned tuna in olive oil	25.33 ± 1.64 ^c^	-	-	-	-	-	-
Canned natural tuna	38.90 ± 1.79 ^d^	-	-	-	13.53 ± 0.21 ^a^	10.01 ± 0.10 ^a^	-
Canned tuna pâté	13.61 ± 0.58 ^b^	7.76 ± 0.25 ^a^	-	-	-	-	-
Natural Alaskan salmon	-	-	-	-	-	-	-
Canned pink salmon	37.62 ± 0.48 ^d^			121.09 ± 0.77 ^c^	54.44 ± 0.47 ^b^	11.78 ± 0.39 ^a^	9.97 ± 0.31 ^a^
Canned sardines in olive oil	2.85 ± 0.50 ^a^	-	-	-	-	-	-
Canned sardines in vegetable oil	7.60 ± 0.39 ^a,b^	-	-	-	-	-	-
Canned crab meat in brine	47.69 ± 0.58 ^d^	10.09 ± 0.19 ^a^	-	28.70 ± 0.36 ^a^	12.44 ± 0.31 ^a^	37.14 ± 0.42 ^b,c^	15.15 ± 0.18 ^b^
Salmon pâté	-	-	-	-	-	-	-
Surimi sticks	20.75 ± 0.68 ^c^	-	-	55.85 ± 0.35 ^b^	12.88 ± 0.24 ^a^	36.71 ± 0.36 ^c^	15.52 ± 0.35 ^b^
Anchovy paste in olive oil	-	-	-	-	-	-	-
Shelled clams	-	-	-	-	-	-	-
Natural shrimp	-	-	-	-	-	-	-
Lumpfish eggs	-	-	-	-	-	-	-
Cuttlefish Ink	-	-	-	-	-	-	-
Dried sardines	3.36 ± 0.39 ^a^	-	-	90.27 ± 0.34 ^b,c^	-	-	-
Dried shrimp	2.68 ± 0.13 ^a^	-	-	-	-	-	-
***p*** **-value**	**0.000**	**0.000**	**-**	**0.000**	**0.000**	**0.000**	**0.000**

Letters ‘a–d’ indicate homogeneous sample groups at α = 0.01, and the same letter indicates samples that are not different. Bold *p*-values indicate significantly different results at *p* < 0.01. Statistical results for BPF, BADGE, BADGE∙2H_2_O, BADGE∙HCl∙H_2_O, and BADGE∙2HCl were obtained considering only positive samples (samples with values lower than LOQ were not entered).

**Table 4:** Bisphenols and BADGE derivates (expressed in µg/kg) of processed meat samples. Data are expressed as mean ± standard deviation, and Kruskal–Wallis test statistics are reported.

Samples	BPA	BPF	BPB	BADGE	BADGE∙ 2H_2_O	BADGE∙HCl∙ H_2_O	BADGE∙ 2HCl
Canned light chicken	20.52 ± 0.64 ^b^	10.35 ± 0.56 ^a^	3.34 ± 0.69	-	-	-	-
Canned halal chicken	-	-	-	-	-	-	-
Canned meat broth	4.30 ± 0.04 ^a^	6.49 ± 0.08 ^a^	-	16.72 ± 0.31 ^a^	9.35 ± 0.08 ^a^	-	-
Canned beef and pork pâté	-	-	-	-	-	-	-
Canned chicken breast with jelly	29.57 ± 1.09 ^c^	11.74 ± 0.29 ^a^	-	23.42 ± 0.27 ^a^	-	-	-
Canned luncheon chicken meat	25.12 ± 0.12 ^b^	-	-	100.62 ± 0.43 ^b^	9.94 ± 0.35 ^a^	10.75 ± 0.22	22.86 ± 0.65
Canned cooked ham	3.28 ± 0.05 ^a^	-	-	-	-	-	-
Canned beef with gelatine	0.52 ± 0.04 ^a^	20.72 ± 0.52 ^b^	-	-	-	-	-
Canned beef and pork pâté	2.75 ± 0.05 ^a^			13.21 ± 0.07 ^a^	10.95 ± 0.09 ^a^		
Canned minced pork and ham with bacon	9.17 ± 0.26 ^a,b^	-	2.34 ± 0.10	151.02 ± 0.57 ^b^	201.90 ± 0.15 ^b^	23.14 ± 0.15	13.81 ± 0.19
***p*** **-value**	**0.000**	**0.000**	**-**	**0.000**	**0.000**	**-**	**0.000**

Letters ‘a–c’ indicate homogeneous sample groups at α = 0.01, and the same letter indicates samples that are not different. Bold *p*-values indicate significantly different results at *p* < 0.01. Statistical results for BPF, BADGE, BADGE∙2H_2_O, BADGE∙HCl∙H_2_O, and BADGE∙2HCl were obtained considering only positive samples (samples with values lower than LOQ were not entered).

**Table 5:** Toxic and potentially toxic elements (expressed in mg/kg) of processed fish samples. Data are expressed as mean ± standard deviation, and Kruskal–Wallis test statistics are reported.

Sample	Zn	As	Cd	Cr	Cu	Fe	Mn	Ni	Pb	Sn	Al
Canned mackerel fillets	9.17 ± 0.31 ^a^	0.50 ± 0.09 ^b^	0.02 ± 0.00 ^a^	0.08 ± 0.01 ^a^	1.59 ± 0.20 ^a,b^	8.87 ± 0.26 ^a^	1.03 ± 0.07 ^b^	0.08 ± 0.02 ^a^	0.07 ± 0.04 ^a^	0.18 ± 0.07 ^a^	4.11 ± 0.54 ^a,b^
Canned horse mackerel	9.48 ± 0.27 ^a^	0.35 ± 0.08 ^a,b^	0.05 ± 0.01 ^a^	0.13 ± 0.02 ^a,b^	1.24 ± 0.12 ^a,b^	8.51 ± 0.30 ^a^	0.97 ± 0.05 ^b^	0.10 ± 0.01 ^a^	0.13 ± 0.0 ^a,b^	0.09 ± 0.01 ^a^	6.57 ± 0.28 ^b^
Grilled mackerel fillets in olive oil	10.39 ± 0.32 ^a,b^	0.28 ± 0.05 ^a,b^	0.02 ± 0.00 ^a^	0.10 ± 0.01 ^a,b^	1.73 ± 0.18 ^a,b^	6.69 ± 0.41 ^a^	0.91 ± 0.04 ^b^	0.05 ± 0.01 ^a^	0.11 ± 0.03 ^a^	0.13 ± 0.03 ^a^	6.66 ± 0.23 ^b^
Canned smoked mackerel fillets	10.81 ± 0.35 ^a,b^	0.34 ± 0.08 ^a,b^	0.01 ± 0.00 ^a^	0.11 ± 0.01 ^a,b^	4.80 ± 0.28 ^b^	14.36 ± 0.99 ^b^	0.90 ± 0.05 ^b^	0.06 ± 0.02 ^a^	0.04 ± 0.01 ^a^	0.59 ± 0.07 ^b^	6.29 ± 0.30 ^b^
Natural grilled mackerel fillets	8.63 ± 0.29 ^a^	0.78 ± 0.05 ^b^	0.04 ± 0.01 ^a^	0.10 ± 0.01 ^a,b^	1.73 ± 0.15 ^a,b^	8.56 ± 0.42 ^a^	0.96 ± 0.06 ^b^	0.05 ± 0.01 ^a^	0.06 ± 0.01 ^a^	0.09 ± 0.02 ^a^	8.54 ± 0.31 ^b^
Mackerel in *sos tomat*	15.33 ± 0.40 ^b^	1.16 ± 0.05 ^c^	0.01 ± 0.00 a	0.13 ± 0.03 ^a,b^	3.91 ± 0.25 ^b^	14.16 ± 0.41 ^b^	1.06 ± 0.06 ^b^	0.09 ± 0.01 ^a^	0.06 ± 0.01 ^a^	0.05 ± 0.01 ^a^	5.58 ± 0.33 ^a,b^
Canned Atlantic mackerel fillets	6.72 ± 0.34 ^a^	0.26 ± 0.04 ^a,b^	0.07 ± 0.03 ^a^	0.07 ± 0.01 ^a^	1.28 ± 0.14 ^a,b^	8.56 ± 0.20 ^a^	1.06 ± 0.05 ^b^	0.06 ± 0.02 ^a^	0.21 ± 0.03 ^a,b^	0.09 ± 0.01 ^a^	7.91 ± 0.14 ^b^
Canned tuna in olive oil	16.30 ± 0.37 ^b^	0.51 ± 0.05 ^b^	0.02 ± 0.01 ^a^	0.14 ± 0.03 ^a,b^	1.41 ± 0.15 ^a,b^	6.29 ± 0.23 ^a^	2.00 ± 0.08 ^c^	0.24 ± 0.04 ^a^	0.09 ± 0.02 ^a^	0.47 ± 0.04 ^a,b^	5.47 ± 0.19 ^a,b^
Canned natural tuna	13.81 ± 0.51 ^b^	0.66 ± 0.08 ^b^	0.03 ± 0.01 ^a^	0.16 ± 0.02 ^a,b^	1.58 ± 0.08 ^a,b^	5.74 ± 0.21 ^a^	1.97 ± 0.07 ^b^	0.29 ± 0.05 ^a^	0.03 ± 0.01 ^a^	0.41 ± 0.05 ^a,b^	4.71 ± 0.15 ^a,b^
Canned tuna pâté	19.47 ± 0.53 ^b,c^	0.84 ± 0.06 ^b^	0.02 ± 0.01 ^a^	0.14 ± 0.03 ^a,b^	1.52 ± 0.12 ^a,b^	5.44 ± 0.38 ^a^	2.10 ± 0.07 ^c^	0.27 ± 0.04 ^a^	0.04 ± 0.01 ^a^	0.44 ± 0.03 ^a,b^	5.15 ± 0.08 ^a,b^
Natural Alaskan salmon	6.29 ± 0.27 ^a^	0.07 ± 0.01 ^a^	0.02 ± 0.01 ^a^	0.05 ± 0.01 ^a^	1.70 ± 0.04 ^a,b^	5.66 ± 0.45 ^a^	0.06 ± 0.01 ^a^	0.20 ± 0.02 ^a^	0.11 ± 0.01 ^a,b^	0.84 ± 0.04 ^b^	3.10 ± 0.12 ^a,b^
Canned pink salmon	6.74 ± 0.29 ^a^	0.09 ± 0.01 ^a^	0.02 ± 0.01 ^a^	0.05 ± 0.02 ^a^	1.71 ± 0.06 ^a,b^	7.10 ± 0.22 ^a^	0.05 ± 0.01 ^a^	0.23 ± 0.02 ^a^	0.18 ± 0.02 ^a,b^	0.82 ± 0.03 ^b^	3.12 ± 0.10 ^a,b^
Canned sardines in olive oil	26.19 ± 1.05 ^c^	0.14 ± 0.01 ^a^	0.13 ± 0.02 ^b^	0.06 ± 0.01 ^a^	1.60 ± 0.22 ^a,b^	20.85 ± 1.19 ^b^	1.27 ± 0.17 ^b^	0.79 ± 0.08 ^b^	0.14 ± 0.03 ^a,b^	0.27 ± 0.02 ^a,b^	4.00 ± 0.09 ^a,b^
Canned sardines in vegetable oil	22.83 ± 0.51 ^c^	0.11 ± 0.02 ^a^	0.13 ± 0.02 ^b^	0.05 ± 0.01 ^a^	1.12 ± 0.10 ^a,b^	20.50 ± 1.01 ^b^	1.14 ± 0.09 ^b^	0.96 ± 0.06 ^b^	0.11 ± 0.02 ^a,b^	0.34 ± 0.02 ^a,b^	3.64 ± 0.18 ^a,b^
Canned crab meat in brine	10.58 ± 0.89 ^a,b^	0.30 ± 0.03 ^a,b^	0.05 ± 0.01 ^a^	0.10 ± 0.01 ^a,b^	0.46 ± 0.09 ^a^	9.63 ± 0.75 ^a,b^	1.55 ± 0.05 ^b^	0.21 ± 0.02 ^a^	0.08 ± 0.01 ^a^	0.75 ± 0.05 ^b^	1.97 ± 0.05 ^a^
Salmon pâté	7.33 ± 0.38 ^a^	0.07 ± 0.01 ^a^	0.01 ± 0.00 ^a^	0.05 ± 0.02 ^a^	1.86 ± 0.05 ^a,b^	5.71 ± 0.33 ^a^	0.08 ± 0.02 ^a^	0.17 ± 0.03 ^a^	0.21 ± 0.02 ^a,b^	0.76 ± 0.05 ^b^	3.37 ± 0.16 ^a,b^
Surimi sticks	4.36 ± 0.18 ^a^	0.41 ± 0.08 ^b^	0.08 ± 0.02 ^a^	0.13 ± 0.03 ^a,b^	0.36 ± 0.05 ^a^	3.90 ± 0.25 ^a^	0.16 ± 0.03 ^a^	0.07 ± 0.02 ^a^	0.10 ± 0.02 ^a^	0.08 ± 0.01 ^a^	0.52 ± 0.05 ^a^
Anchovy paste in olive oil	21.00 ± 1.15 ^c^	1.42 ± 0.24 ^c^	0.17 ± 0.04 ^b^	0.06 ± 0.01 ^a^	3.31 ± 0.19 ^b^	33.17 ± 1.98 ^c^	1.22 ± 0.06 ^b^	0.12 ± 0.03 ^a^	0.13 ± 0.04 ^a,b^	0.13 ± 0.02 ^a^	7.68 ± 0.23 ^b^
Shelled clams	20.27 ± 0.69 ^c^	0.85 ± 0.06 ^b^	0.03 ± 0.01 ^a^	0.39 ± 0.04 ^b^	0.68 ± 0.07 ^a^	70.41 ± 0.82 ^d^	0.68 ± 0.08 ^a^	0.25 ± 0.04 ^a^	0.09 ± 0.01 ^a^	0.12 ± 0.02 ^a^	1.61 ± 0.10 ^a^
Natural shrimp	4.36 ± 0.67 ^a^	0.34 ± 0.05 ^a,b^	0.03 ± 0.01 ^a^	0.12 ± 0.03 ^a,b^	8.35 ± 0.37 ^b^	15.98 ± 0.23 ^b^	1.06 ± 0.07 ^b^	3.48 ± 0.22 ^c^	0.09 ± 0.02 ^a^	0.04 ± 0.01 ^a^	0.55 ± 0.09 ^a^
Lumpfish eggs	11.32 ± 0.21 ^b^	0.23 ± 0.03 ^a^	0.04 ± 0.01 ^a^	0.10 ± 0.02 ^a,b^	0.86 ± 0.06 ^a^	4.76 ± 0.20 ^a^	0.36 ± 0.05 ^a^	0.20 ± 0.02 ^a^	0.08 ± 0.01 ^a^	0.05 ± 0.01 ^a^	0.37 ± 0.05 ^a^
Cuttlefish ink	17.89 ± 0.81 ^b^	0.49 ± 0.06 ^b^	0.91 ± 0.05 ^c^	0.10 ± 0.02 ^a,b^	4.58 ± 0.38 ^b^	5.45 ± 0.29 ^a^	0.31 ± 0.04 ^a^	0.14 ± 0.03 ^a^	0.31 ± 0.04 ^b^	0.05 ± 0.01 ^a^	1.11 ± 0.18 ^a^
Dried sardines	23.59 ± 1.43 ^c^	0.14 ± 0.02 ^a^	0.19 ± 0.03 ^b^	0.12 ± 0.03 ^a,b^	2.55 ± 0.23 ^b^	30.18 ± 1.84 ^c^	2.46 ± 0.28 ^c^	1.78 ± 0.18 ^b^	0.15 ± 0.02 ^a,b^	0.10 ± 0.02 ^a^	2.04 ± 0.13 ^a,b^
Dried shrimp	9.54 ± 0.40 ^a^	0.63 ± 0.06 ^b^	0.07 ± 0.01 ^a^	0.20 ± 0.02 ^a,b^	13.46 ± 0.47 ^c^	20.69 ± 0.81 ^b^	1.84 ± 0.12 ^b^	4.22 ± 0.25 ^c^	0.14 ± 0.04 ^a,b^	0.10 ± 0.02 ^a^	0.10 ± 0.02 ^a^
***p*** **-value**	**0.000**	**0.000**	**0.000**	**0.000**	**0.000**	**0.000**	**0.000**	**0.000**	**0.000**	**0.000**	**0.000**

Letters ‘a–c’ indicate homogeneous sample groups at α = 0.01, and the same letter indicates samples that are not different. Bold *p*-values indicate significantly different results at *p* < 0.01.

**Table 6:** Toxic and potentially toxic elements (expressed in mg/kg) of processed meat samples. Data are expressed as mean ± standard deviation, and Kruskal–Wallis test statistics are reported.

Sample	Zn	As	Cd	Cr	Cu	Fe	Mn	Ni	Pb	Sn	Al
Canned light chicken	10.29 ± 0.14 ^a,b^	-	0.02 ± 0.00 ^a^	0.16 ± 0.02 ^a^	0.72 ± 0.06 ^a^	10.56 ± 0.33 ^a^	0.70 ± 0.03 ^b^	0.02 ± 0.01 ^a^	0.07 ± 0.01 ^a^	0.16 ± 0.02 ^a^	0.82 ± 0.06 ^a^
Canned halal chicken	9.59 ± 0.33 ^a^	-	0.03 ± 0.01 ^a^	0.19 ± 0.02 ^a^	0.55 ± 0.05 ^a^	11.23 ± 0.22 ^a^	0.77 ± 0.03 ^b^	0.04 ± 0.01 ^b^	0.16 ± 0.02 ^a^	0.51 ± 0.05 ^a,b^	3.59 ± 0.11 ^b,c^
Canned meat broth	19.54 ± 0.59 ^b^	0.02 ± 0.01	0.04 ± 0.01 ^b^	0.10 ± 0.02 ^a^	1.77 ± 0.21 ^b^	12.93 ± 0.34 ^a^	0.39 ± 0.03 ^a^	0.02 ± 0.01 ^a^	0.20 ± 0.02 ^a^	0.33 ± 0.03 ^a^	0.84 ± 0.06 ^a^
Canned beef and pork pâté	16.04 ± 0.67 ^b^	-	0.01 ± 0.00 ^a^	0.24 ± 0.02 ^b^	0.95 ± 0.05 ^a^	21.23 ± 0.33 ^a,b^	0.19 ± 0.01 ^a^	0.01 ± 0.00 ^a^	0.28 ± 0.02 ^b^	0.05 ± 0.01 ^a^	0.95 ± 0.07 ^a^
Canned chicken breast with jelly	11.87 ± 0.44 ^a,b^	-	0.05 ± 0.01 ^b^	0.25 ± 0.02 ^b^	0.79 ± 0.03 ^a^	12.54 ± 0.43 ^a^	0.94 ± 0.04 ^b^	0.05 ± 0.01 ^a^	0.08 ± 0.01 ^a^	0.35 ± 0.06 ^a^	2.03 ± 0.18 ^b^
Canned luncheon chicken meat	9.40 ± 0.36 ^a^	0.29 ± 0.02	0.05 ± 0.01 ^b^	0.20 ± 0.01 ^a^	0.60 ± 0.06 ^a^	17.24 ± 0.16 ^a,b^	0.65 ± 0.04 ^a,b^	0.02 ± 0.01 ^a^	0.20 ± 0.01 ^a^	1.69 ± 0.12 ^b^	4.90 ± 0.14 ^c^
Canned cooked ham	6.87 ± 0.19 ^a^	0.06 ± 0.01	0.04 ± 0.01 ^b^	0.13 ± 0.02 ^a^	1.23 ± 0.11 ^b^	13.45 ± 0.65 ^a^	0.54 ± 0.03 ^a^	0.02 ± 0.00 ^a^	0.20 ± 0.02 ^a^	0.24 ± 0.02 ^a^	0.61 ± 0.06 ^a^
Canned beef with gelatine	7.60 ± 0.06 ^a^	0.02 ± 0.01	0.03 ± 0.01 ^a^	0.30 ± 0.04 ^b^	0.81 ± 0.05 ^a^	27.30 ± 0.22 ^b^	0.23 ± 0.02 ^a^	0.05 ± 0.01 ^b^	0.20 ± 0.03 ^a^	0.22 ± 0.04 ^a^	0.54 ± 0.08 ^a^
Canned beef and pork pâté	16.38 ± 0.42 ^b^	-	0.02 ± 0.01 ^a^	0.27 ± 0.02 ^b^	1.04 ± 0.08 ^a,b^	21.95 ± 0.83 ^a,b^	0.22 ± 0.03 ^a^	0.02 ± 0.01 ^a^	0.15 ± 0.02 ^a^	0.90 ± 0.09 ^a,b^	1.05 ± 0.07 ^b^
Canned minced pork and ham with bacon	9.46 ± 0.31 ^a^	-	0.10 ± 0.02 ^a^	0.29 ± 0.03 ^b^	1.54 ± 0.23^b^	13.11 ± 0.88 ^a^	0.31 ± 0.03 ^a^	0.01 ± 0.00 ^a^	0.09 ± 0.01 ^a^	0.22 ± 0.04 ^a^	2.14 ± 0.11 ^b^
***p*** **-value**	**0.000**	**-**	**0.000**	**0.000**	**0.000**	**0.000**	**0.000**	**0.000**	**0.000**	**0.000**	**0.000**

Letters ‘a–c’ indicate homogeneous sample groups at α = 0.01, and the same letter indicates samples that are not different. Bold *p*-values indicate significantly different results at *p* < 0.01.

**Table 7:** Estimated weekly intake (EWI) and hazard quotient (HQ) calculated for the processed fish and meat samples investigated.

Sample	BPA		Zn		As		Cd		Cu		Mn		Ni		Pb		Al	
	TDI (ng/kg)		PTWI (µg/kg)		PTWI (µg/kg)		TWI (µg/kg)		PTWI (µg/kg)		PTWI (µg/kg)		TDI (µg/kg)		BMDL01 (µg/kg)		TWI (µg/kg)	
	EWI	HQ	EWI	HQ	EWI	HQ	EWI	HQ	EWI	HQ	EWI	HQ	EWI	HQ	EWI	HQ	EWI	HQ
Canned mackerel fillets	43.62	31.16	19.64	<1	1.08	<1	0.05	<1	3.41	<1	2.21	<1	0.16	<1	0.15	<1	8.81	<1
Canned horse mackerel	9.94	7.10	21.66	<1	0.79	<1	0.10	<1	2.82	<1	2.22	<1	0.22	<1	0.30	<1	15.01	<1
Grilled mackerel fillets in olive oil	116.62	83.30	35.63	<1	0.97	<1	0.08	<1	5.93	<1	3.12	<1	0.17	<1	0.36	<1	22.83	<1
Canned smoked mackerel fillets	72.80	52.00	24.70	<1	0.77	<1	0.02	<1	10.97	<1	2.05	<1	0.14	<1	0.08	<1	14.37	<1
Natural grilled mackerel fillets	26.61	19.01	24.67	<1	2.23	<1	0.12	<1	4.94	<1	2.74	<1	0.13	<1	0.17	<1	24.40	<1
Mackerel in *sos tomat*	27.64	19.74	35.03	<1	2.65	<1	0.02	<1	8.93	<1	2.42	<1	0.21	<1	0.14	<1	12.76	<1
Canned Atlantic mackerel fillets	-	-	15.35	<1	0.59	<1	0.17	<1	2.93	<1	2.42	<1	0.13	<1	0.47	<1	18.07	<1
Canned tuna in olive oil	57.90	41.36	37.26	<1	1.17	<1	0.05	<1	3.21	<1	4.57	<1	0.55	<1	0.21	<1	12.50	<1
Canned natural tuna	88.92	63.51	31.55	<1	1.51	<1	0.06	<1	3.60	<1	4.50	<1	0.66	<1	0.06	<1	10.77	<1
Canned tuna pâté	31.11	22.22	44.51	<1	1.93	<1	0.03	<1	3.47	<1	4.79	<1	0.61	<1	0.10	<1	11.78	<1
Natural Alaskan salmon	-	-	14.37	<1	0.17	<1	0.05	<1	3.89	<1	0.13	<1	0.45	<1	0.24	<1	7.09	<1
Canned pink salmon	85.98	61.41	15.41	<1	0.21	<1	0.04	<1	3.90	<1	0.11	<1	0.52	<1	0.42	<1	7.12	<1
Canned sardines in olive oil	7.33	5.23	67.33	<1	0.36	<1	0.33	<1	4.11	<1	3.26	<1	2.04	<1	0.36	<1	10.29	<1
Canned sardines in vegetable oil	17.38	12.41	52.18	<1	0.24	<1	0.29	<1	2.55	<1	2.61	<1	2.20	<1	0.24	<1	8.33	<1
Canned crab meat in brine	109.01	77.86	24.18	<1	0.69	<1	0.12	<1	1.05	<1	3.53	<1	0.48	<1	0.18	<1	4.50	<1
Salmon pâté	-	-	0.00	<1	0.00	<1	0.00	<1	0.00	<1	0.00	<1	0.00	<1	0.00	<1	0.00	<1
Surimi sticks	59.28	42.34	12.44	<1	1.18	<1	0.23	<1	1.04	<1	0.44	<1	0.19	<1	0.27	<1	1.49	<1
Anchovy paste in olive oil	-	-	47.99	<1	3.25	<1	0.39	<1	7.56	<1	2.79	<1	0.27	<1	0.30	<1	17.55	<1
Shelled clams	-	-	46.34	<1	1.93	<1	0.07	<1	1.55	<1	1.55	<1	0.57	<1	0.20	<1	3.67	<1
Natural shrimp	-	-	9.97	<1	0.78	<1	0.07	<1	19.09	<1	2.42	<1	7.96	<1	0.20	<1	1.25	<1
Lumpfish eggs	-	-	0.00	<1	0.00	<1	0.00	<1	0.00	<1	0.00	<1	0.00	<1	0.00	<1	0.00	<1
Cuttlefish ink	-	-	0.00	<1	0.00	<1	0.00	<1	0.00	<1	0.00	<1	0.00	<1	0.00	<1	0.00	<1
Dried sardines	7.68	5.49	53.92	<1	0.32	<1	0.43	<1	5.84	<1	5.61	<1	4.06	<1	0.34	<1	4.66	<1
Dried shrimp	6.13	4.38	21.81	<1	1.44	<1	0.16	<1	30.76	<1	4.21	<1	9.64	<1	0.32	<1	0.23	<1
Canned light chicken	46.90	33.50	23.51	<1	-	-	0.05	<1	1.65	<1	1.61	<1	0.04	<1	0.15	<1	1.88	<1
Canned halal chicken	-	-	21.91	<1	-	-	0.08	<1	1.26	<1	1.77	<1	0.08	<1	0.37	<1	8.21	<1
Canned meat broth	9.82	7.01	44.66	<1	0.04	<1	0.10	<1	4.05	<1	0.89	<1	0.04	<1	0.46	<1	1.92	<1
Canned beef and pork pâté	-	-	36.66	<1	-	-	0.02	<1	2.17	<1	0.44	<1	0.02	<1	0.64	<1	2.17	<1
Canned chicken breast with jelly	67.58	48.27	27.14	<1	-	-	0.11	<1	1.80	<1	2.15	<1	0.11	<1	0.19	<1	4.63	<1
Canned lunchon chicken meat	57.42	41.02	21.49	<1	0.66	<1	0.11	<1	1.38	<1	1.49	<1	0.04	<1	0.46	<1	11.21	<1
Canned cooked ham	7.49	5.35	15.70	<1	0.13	<1	0.10	<1	2.81	<1	1.23	<1	0.05	<1	0.45	<1	1.39	<1
Canned beef with gelatine	1.20	<1	17.37	<1	0.05	<1	0.06	<1	1.85	<1	0.52	<1	0.11	<1	0.46	<1	1.23	<1
Canned beef and pork pâté	6.29	4.50	37.43	<1	-	-	0.04	<1	2.37	<1	0.50	<1	0.04	<1	0.35	<1	2.40	<1
Canned minced pork and ham with bacon	20.97	14.98	21.62	<1	-	-	0.22	<1	3.53	<1	0.70	<1	0.02	<1	0.21	<1	4.89	<1

## Data Availability

The original contributions presented in the study are included in the article/Supplementary Materials; further inquiries can be directed to the corresponding author.

## Supplementary Materials

The following supporting information can be downloaded at https://www.mdpi.com/article/10.3390/toxics13060433/s1, Table S1: Analytical validation of HPLC-MS/MS method; Table S2: Analytical validation of ICP-MS method. Figure S1: Chromatogram of the canned tuna pate sample. Figure S2: ESI mass spectrum of the canned tuna pate sample.
