# Supplementary material for: Bisphenols, Toxic Elements, and Potentially Toxic Elements in Ready-to-Eat Fish and Meat Foods and Their Associated Risks for Human Health
Source: Toxics. 2025 May 25;13(6):433. doi: 10.3390/toxics13060433 (PMC12196756; doi:10.3390/toxics13060433)
Supplement: Supplementary file 1 [file toxics-13-00433-s001.zip › toxics-3596347-supplementary.pdf]

Supporting Information

Table S1. Analytical validation of HPLC-MS/MS method.

Analyte	Abbreviation	Precursor ion (m/z)	Product Ions (m/z) Quantification	Product Ions (m/z) Confirmation	R^2	LOD (µg/kg)	LOQ (µg/kg)
		$[M-H]^-$					
2,2-bis-(4-hydroxyphenyl)-propane	BPA	227.29	227.3 → 212.1	227.3 → 133.0	0.9998	0.4	1.3
4,4′-Sulfonyldiphenol	BPS	249.27	249.3 → 107.9	249.3 → 156.0	0.9996	0.5	1.7
4,4′-Methylenediphenol	BPF	199.24	199.2 → 93.1	199.2 → 105.1	0.9994	1.5	5.0
4,4′-sec-Butylidenediphenol	BPB	241.31	241.3 → 212.0	241.3 → 211.0	0.9996	0.5	1.7
4,4′-Ethylidenebisphenol	BPE	213.26	213.3 → 198.0	213.3→194.9	0.9991	3.0	10.0
4,4′-Cyclohexylidenebisphenol	BPZ	267.35	267.3 → 145.0	267.3 → 173.1	0.9989	0.5	1.7
4,4′-(1,4-Phenylenediisopropylidene) bisphenol	BPP	345.47	345.5 → 330.1	345.5 → 133.1	0.9992	1.5	5.0
4,4′-(1-Phenylethylidene) bisphenol	BPAP	289.36	289.4 → 274.1	289.4 → 273.1	0.9995	1.5	5.0
4,4′-(Hexafluoroisopropylidene)diphenol	BPAF	335.24	335.3 → 265.0	335.3 → 177.0	0.9996	1.0	3.3
		$[M+NH_4]^+$					
Bisphenol A bis(2,3-dihydroxypropyl) ether	BADGE·$2H_2O$	394.2	394.2 → 209.1	394.2 → 135.1	0.9987	2.5	8.3
Bisphenol A (3-chloro-2-hydroxypropyl) (2,3-dihydroxypropyl) ether	BADGE·HCl·H_2O	412.2	412.2 → 227.0	412.2 → 135.1	0.9985	0.5	1.7
Bisphenol A bis(3-chloro-2-hydroxypropyl) ether	BADGE·2HCl	430.2	430.2 → 227.1	430.2 → 135.2	0.9988	0.8	2.7
Bisphenol A diglycidyl ether	BADGE	358.2	358.2 → 191.0	358.2 → 135.1	0.9990	3.0	10.0
Bisphenol A dimethacrylate	BPADMA	382.2	382.2 → 323.9	382.2 → 189.8	0.9982	2.5	8.3
Bisphenol F bis (2,3-dihydroxypropyl) ether	BFDGE·$2H_2O$	366.2	366.2 → 133.1	366.2 → 181.1	0.9984	2.5	8.3

Table S2. Analytical validation of ICP-MS method.

Element	LOD (mg/kg)	LOQ (mg/kg)	R2	ERM-CE278k Muscle tissue (%)	ERMBB184 Bovine muscle (%)
Al	0.010	0.033	0.9995	96.77 ± 0.68*	97.17 ± 0.89*
As	0.001	0.003	0.9997	98.20 ± 1.05	97.55 ± 0.75
Cd	0.001	0.003	0.9999	101.75 ± 1.15	102.50 ± 1.23
Cr	0.001	0.003	0.9995	98.15 ± 0.72	97.75 ± 0.74*
Cu	0.004	0.013	0.9994	97.10 ± 1.06	97.65 ± 1.18
Fe	0.013	0.043	0.9997	98.15 ± 0.95	97.85 ± 0.88
Mn	0.003	0.010	0.9996	97.60 ± 0.84	97.23 ± 0.46
Ni	0.001	0.003	0.9997	97.75 ± 0.93	97.33 ± 0.76
Pb	0.001	0.003	0.9999	104.45 ± 1.01	105.1 ± 1.35
Sn	0.003	0.010	0.9994	96.55 ± 0.76*	96.25 ± 0.53*
Zn	0.020	0.066	0.9996	98.50 ± 0.95	98.10 ± 0.77

* Analytes not included in the certified matrix.

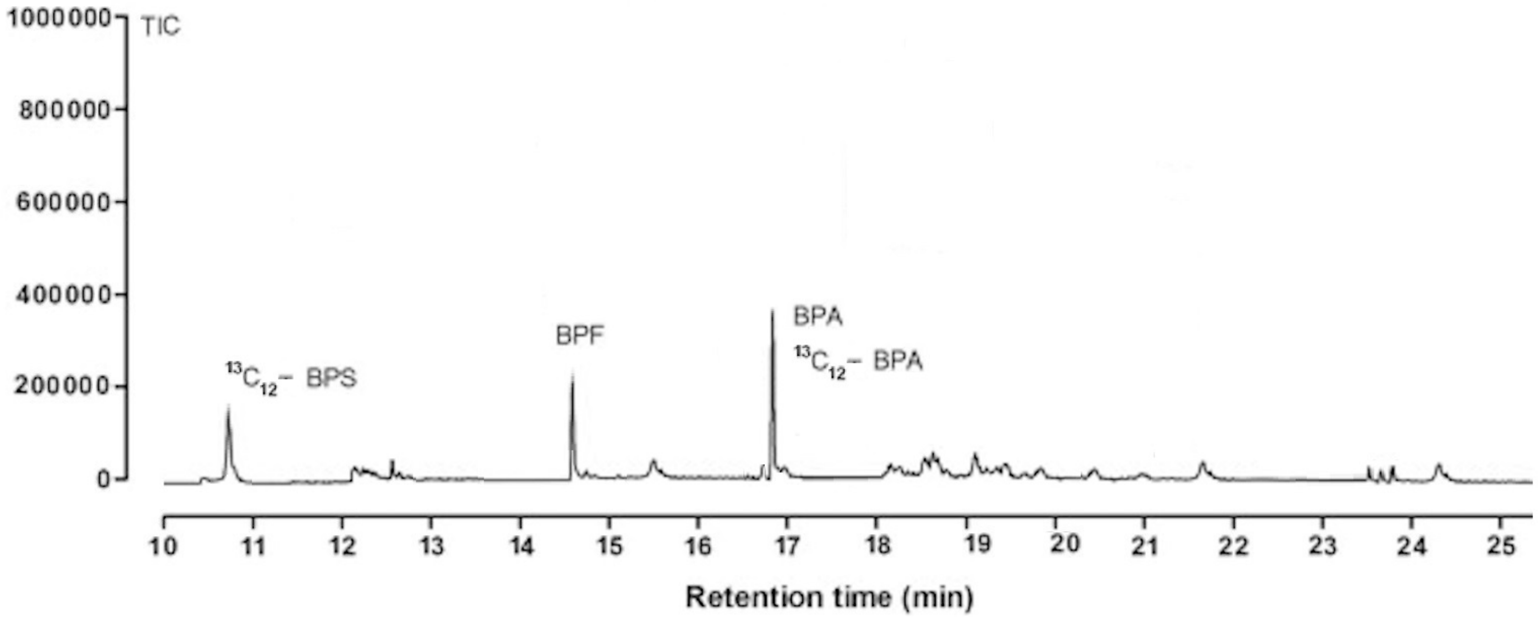

Figure S1: Chromatogram of the canned tuna pate sample.

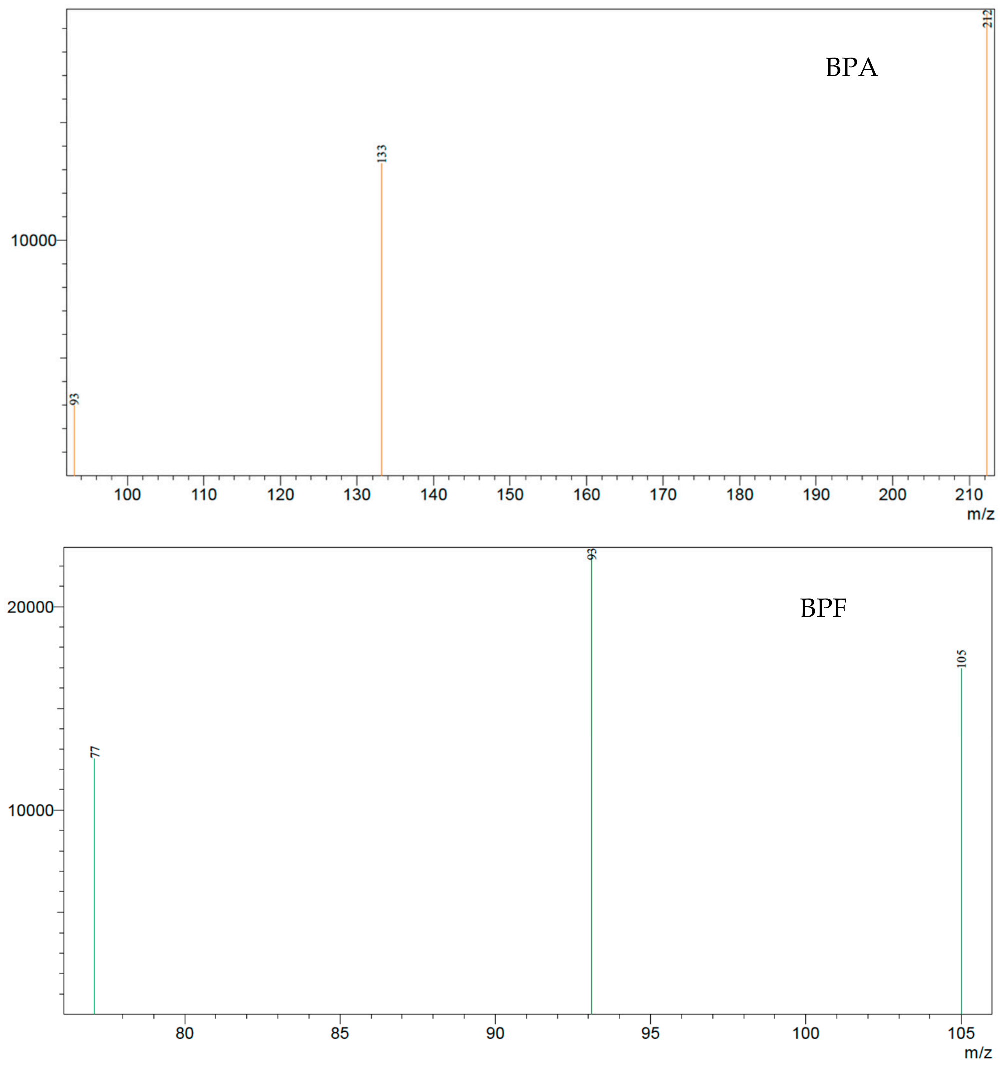

Figure S2: ESI mass spectrum of the canned tuna pate sample.
